# Cyclical and Patch-Like GDNF Distribution along the Basal Surface of Sertoli Cells in Mouse and Hamster Testes

**DOI:** 10.1371/journal.pone.0028367

**Published:** 2011-12-09

**Authors:** Takeshi Sato, Yoshimi Aiyama, Mayuko Ishii-Inagaki, Kenshiro Hara, Naoki Tsunekawa, Kyoko Harikae, Mami Uemura-Kamata, Mai Shinomura, Xiao Bo Zhu, Seishi Maeda, Sachi Kuwahara-Otani, Akihiko Kudo, Hayato Kawakami, Masami Kanai-Azuma, Michio Fujiwara, Yoichi Miyamae, Shosei Yoshida, Makoto Seki, Masamichi Kurohmaru, Yoshiakira Kanai

**Affiliations:** 1 Department of Veterinary Anatomy, The University of Tokyo, Tokyo, Japan; 2 Drug Safety Research Labs, Astellas Pharma Inc., Osaka, Japan; 3 Division of Germ Cell Biology, National Institute for Basic Biology and Department of Basic Biology, School of Life Science, Graduate University for Advanced Studies (SOKENDAI), Okazaki, Japan; 4 Center for Experimental Animal, Tokyo Medical and Dental University, Tokyo, Japan; 5 Division of Cell Biology, Department of Anatomy, Hyogo College of Medicine, Nishinomiya, Japan; 6 Department of Anatomy, Kyorin University School of Medicine, Mitaka, Japan; National Cancer Center, Japan

## Abstract

**Background and Aims:**

In mammalian spermatogenesis, glial cell line-derived neurotrophic factor (GDNF) is one of the major Sertoli cell-derived factors which regulates the maintenance of undifferentiated spermatogonia including spermatogonial stem cells (SSCs) through GDNF family receptor α1 (GFRα1). It remains unclear as to when, where and how GDNF molecules are produced and exposed to the GFRα1-positive spermatogonia in vivo.

**Methodology and Principal Findings:**

Here we show the cyclical and patch-like distribution of immunoreactive GDNF-positive signals and their close co-localization with a subpopulation of GFRα1-positive spermatogonia along the basal surface of Sertoli cells in mice and hamsters. Anti-GDNF section immunostaining revealed that GDNF-positive signals are mainly cytoplasmic and observed specifically in the Sertoli cells in a species-specific as well as a seminiferous cycle- and spermatogenic activity-dependent manner. In contrast to the ubiquitous GDNF signals in mouse testes, high levels of its signals were cyclically observed in hamster testes prior to spermiation. Whole-mount anti-GDNF staining of the seminiferous tubules successfully visualized the cyclical and patch-like extracellular distribution of GDNF-positive granular deposits along the basal surface of Sertoli cells in both species. Double-staining of GDNF and GFRα1 demonstrated the close co-localization of GDNF deposits and a subpopulation of GFRα1-positive spermatogonia. In both species, GFRα1-positive cells showed a slender bipolar shape as well as a tendency for increased cell numbers in the GDNF-enriched area, as compared with those in the GDNF-low/negative area of the seminiferous tubules.

**Conclusion/Significance:**

Our data provide direct evidence of regionally defined patch-like GDNF-positive signal site in which GFRα1-positive spermatogonia possibly interact with GDNF in the basal compartment of the seminiferous tubules.

## Introduction

In mammalian testes, spermatogonial stem cells (SSCs) are continuously maintained by self-renewal in the basal compartment of seminiferous tubules. This compartment is defined as the area between the tight junction of Sertoli cells and the continuous basal lamina [1]–[4]. SSCs are a small subset of spermatogonia that express GFRα1 (a GPI-linked cell surface protein) and Nanos2 (a zinc-finger RNA-binding protein), and are mostly A_single_ (singly isolated) and A_paired_ (two interconnected cells) cells [5]–[7]. GFRα1-positive cells then give rise to longer spermatogonial chain (A_aligned_ [chains of 4, 8, 16 and 32 cells etc.]), which then differentiate into a larger number of advanced spermatogenic cells during the basal-to-adluminal translocation of the seminiferous epithelium [8]–[10]. These cells ultimately form spermatozoa at the luminal edge.

In most mammals, it is likely that the balance between self-renewal and differentiation in the SSCs pool is primarily regulated by glial cell line-derived neurotrophic factor (GDNF) which is produced by Sertoli cells [3], [11]–[13]. In *Gdnf*-heterozygote mice, the undifferentiated spermatogonia including SSCs are reduced in number in the basal compartment of the seminiferous tubules, resulting in a lack of spermatogenic cells from the basal to apical side in older mice [11]. Moreover, in *Gdnf*-overexpressing mice, the SSC-like cells are clearly increased in number in the basal region, leading to defective spermatogonial differentiation without any spermatozoa [11]. It has also been shown that GDNF is essential for the maintenance of the SSC self-renewal in vitro (germ line stem [GS] cells) [14]–[16]. SSCs, with both self-renewal and differentiation capabilities, can be maintained in serum-free conditions with GDNF and several other factors including bFGF and EGF [14]–[18]. Moreover, GDNF enhances the short-term proliferation and survival of bovine SSCs [19]–[21] and the long-term expansion of hamster SSCs [22] in vitro. These data indicate that, in mammalian spermatogenesis, GDNF is one of the major regulators which control the maintenance of the SSC pool in a dose-dependent manner.

In A_single_ and A_paired_ spermatogonia, including the SSC pool, it has been shown that GFRα1/c-Ret mediates secreted GDNF signals to involve in regulation of their proliferation and survival [12], [23], [24] positively through Srk and AKT signaling [25]–[27] and negatively through PLZF-derived mTOR signaling [28]. On the other hand, it is likely that *Gdnf* mRNA expression is also regulated in Sertoli cells in relation to their spermatogenic activities. For example, *Gdnf* expression level is up-regulated in *W/W^v^* testes which lack spermatogenic cells due to a germ cell-autonomous defect, as compared with that in normal testes [29]. This up-regulation is possibly a positive response to compensate for the reduction in the number of germ cells in the tubules. It has also been shown that *Gdnf* expression can be up-regulated by pituitary follicle stimulating hormone, FSH [29], [30]. This finding suggests a possible mechanism for the control of SSC self-renewal at the hypothalamic-pituitary-gonadal axis, especially in seasonal breeders like hamster and some domestic animals (e.g., horse, sheep, and goat). Moreover, these findings suggest that finely-tuned control of the level of GDNF expression is crucial for the maintenance of a constant number of SSCs which, in turn, leads to normal spermatogenesis and fertility. It remains unclear, however, as to when and where GDNF molecules are produced, secreted, and exposed to the GFRα1-positive spermatogonia in the basal compartment of seminiferous epithelia in vivo.

In this study, we demonstrated the spatiotemporal patterns of immunoreactive GDNF molecules in mouse and hamster testes in active and inactive states to identify a potential interaction site between GDNF molecules and GFRα1-positive spermatogonia. Here, we showed the cyclical and patch-like distribution of immunoreactive granular GDNF deposits along the basal surface of Sertoli cells and their close co-localization with a subpopulation of GFRα1-positive spermatogonia in vivo.

## Materials and Methods

### Ethics statement

All animal experiments in this study were carried out in strict accordance with the recommendations in accordance with the Guidelines for Animal Use and Experimentation as set out by the University of Tokyo. The procedures were approved by the Institutional Animal Care and Use Committee of the graduate school of agricultural and life sciences in the University of Tokyo, and the approval IDs are P11-500 and P11-503.

### Animals

ICR, C57BL6, *W/W^v^* and Green (B6-Tg(CAG-EGFP) mice (8-week-old; SLC, Japan) and Syrian hamsters (8-week-old; Nisseiken and SLC, Japan) were used in this study (more than four animals were examined for each experiment group). Antibody specificity was confirmed using testes obtained from three *Gdnf*-null newborn pups, a mutant line known for postnatal lethality [31]. Gonadally inactive (i.e., photoinhibited and hibernating) testes were prepared by transferring male hamsters (8-week-old; total 36 animals) from a long (conventional) photoperiod (14 h L, 10 h D) to a short photoperiod (6 h L, 18 h D), as described previously [32]. Then, at Week 13 at the peak of testicular photoregression, approximately half of the animals were transferred from an ambient temperature of 23°C to 5°C to induce hibernation 4 to 8 weeks later.

### Preparation of W/W^v^ Testes Transplanted with Spermatogonial Stem Cells

For spermatogonial transplantation, cell suspensions including spermatogonia were prepared from 10-day-old wild-type and Green C57BL6 males and transplanted into the testes of 8-week-old recipient *W/W^v^* mice (total 6 recipient males), as described previously [33]–[35]. At 3 months after transplantation, the recipient *W/W^v^* testes were dissected and processed for immunohistochemical analyses.

### Immunohistochemistry

For section immunohistochemical staining, testes were isolated at various stages, and fixed in Bouin's solution or 4% PFA for 4 h. The specimens were dehydrated in ethanol, cleared in xylene, and then routinely embedded in paraffin. The deparaffinized sections were incubated with rabbit anti-GDNF (1∶200 dilution; sc-328, against the sequences within amino acids 155–205 of GDNF [protein accession P39905]; Santa Cruz Biotechnology) antibody at 4°C for 12 h. The reaction was visualized with biotin-conjugated secondary antibody in combination with Elite ABC kit (Vector Laboratories, CA). Some sections stained with anti-GDNF antibody were re-stained with periodic acid Schiff (PAS) reagent to accurately stage the seminiferous cycle. In the immunostained testicular samples of hamsters exposed to a short photoperiod/low ambient temperature, we counted the number of seminiferous tubules with or without GDNF-positive signals, and then estimated the relative incidence of GDNF-positive seminiferous epithelia at each stage. Immunohistochemical staining in each sample was conducted at least three times to confirm its reproducibility.

For whole-mount immunohistochemistry without permeabilization, all testes (16 and 28 testes used in hamsters and mice, respectively) were removed from the tunica albuginea and dispersed roughly in cold PBS. The tissues were then fixed in 4% PFA for 8 to 12 h at 4°C and washed with cold PBS several times. Without any permeabilization steps using methanol and detergent (Tween20/Triton X-100), the seminiferous tubule fragments were incubated with rabbit anti-GDNF (1∶200 dilution) and goat anti-GFRα1 (1∶100 dilution; R&D Systems)/goat anti-mouse c-kit (1∶100 dilution in mice, 1∶20 in hamsters; R&D Systems) antibodies at 4°C for 12 h. After being washed with PBS, the samples were incubated with Alexa-488/594 conjugated secondary antibodies, including DAPI, at room temperature for 2 h. After counter-staining with DAPI, the samples were analyzed under Olympus fluorescent microscope (BX51N-34-FL2) and stereomicroscope (SZX16 plus U-LH100HG) systems and Olympus FluoView confocal laser microscope (FV10i; Olympus, Japan) in combination with Volocity software (Mitani Sangyo, Japan). Whole-mount samples stained with anti-GDNF (green) and anti-GFRα1 (red) were photographed (×400) separately in the GDNF-high and GDNF-low/negative regions of the seminiferous tubule, and then the relative cell number of A_single_∼A_aligned_ subpopulations and A_single_ subpopulation per mm^2^ of seminiferous tubule was estimated in each region. Moreover, the lengths of cell processes at both the long (maximum) and short (minimum) axes were separately analyzed in each selected GFRα1-positive cell (i.e., only A_single_ cells that are located near the centerline/away from the shoulder of the whole-mount tubule samples) using a CV-9 Um pen-type map-meter (Koizumi Sokki Mfg, Japan). In some whole-mount stained samples, Z-stack images on the X-Y plane were collected via confocal microscopy, and then three-dimensional reconstructions and their X-Z plane images were analyzed.

For transmission electron microscopy, the PFA-fixed seminiferous tubules were stained with anti-GDNF antibody in combination with HRP-conjugated anti-rabbit antibody as described above. After development with DAB-H_2_O_2_, the samples were re-fixed in 2.5% glutaraldehyde-0.1 M phosphate buffer (PB) at 4°C for 4 h. The samples were postfixed in 1% OsO_4_ at 4°C for 2 h, dehydrated in ethanol, and then embedded in EPON 812. Ultrathin sections were cut, and then observed under a JEM 1010 transmission electron microscope (JEOL, Japan) at 80 kV.

As negative controls, anti-GDNF antibody was pre-incubated with GDNF peptide (sc-328P; Santa Cruz Biotechnology) prior to use in section and whole mount immunostaining.

### In situ Hybridization

Whole-mount and section in situ hybridization were performed as previously described [36]–[38]. The PFA-fixed seminiferous tubules were directly applied for whole mount in situ hybridization, while the deparaffinized sections of the testes fixed in Bouin's solution were used for section in situ hybridization. Hybridization of hamster *Gdnf* was carried out at 68°C for 16 h. Hamster *Gdnf* cDNA fragments were isolated by RT-PCR using mouse and hamster testes cDNA samples, and then subcloned into pGEM-T (Promega) to prepare RNA probes and determine the amino acid sequence of hamster GDNF. The section and whole-mount in situ hybridization staining was conducted four and three times to confirm its reproducibility, respectively.

### Statistical Analysis

Student's t-test was performed for quantitative data of cell number and length of cell processes of GFRα1-positive cells in whole-mount immunostained samples (Table 1). For the relative numbers of the GDNF-positive seminiferous tubules in the immunostained section samples, Dennett test was performed to determine their statistical significance (see Table S1).

**Table 1:** Comparison of quantitative data showing cell morphological parameters (slender shape) and relative cell density of GFRα1-positive spermatogonia between immunoreactive GDNF-high and -low/negative areas of the seminiferous tubule in mice and hamsters1).

	GDNF level		Cell shape parameter (µm)		No. of GFRα1- positive cells (total number of cells counted)2)	
		Long axis	Short axis	Ratio (long/short)		
Hamster	High	53.8±4.2	5.5±0.2	10.4±1.1^a^*	50.1±8.2^c^	(86)
					8.7±2.6^c^	(15)
	Low	44.2±5.4	6.7±0.3	7.1±1.0^a^	43.0±8.3^d^	(82)
					8.4±1.4^d^	(16)
Mouse	High	31.3±2.2	4.6±0.2	7.1±0.7^b^*	179.5±27.1^c^**	(115)
					20.3±3.8^c^*	(13)
	Low	27.3±2.1	5.2±0.2	5.4±0.5^b^	140.4±12.1^d^**	(120)
					15.2±3.0^d^*	(13)

1) Whole seminiferous tubules were double-stained with anti-GDNF (green) and anti-GFRα1 (red), and then photographed (×400). Both the length of the cell process (at long [maximum radius] and short [minimum radius] axes) and relative cell shape (long/short value) of GFRα1-positive cells (only A_single_ type) in GDNF-high and -low/negative areas of the seminiferous tubule were separately calculated. Moreover, the relative number of GFRα1-positive cells (A_single_∼A_aligned_ types) was separately estimated in GDNF-high and -low/negative areas of the seminiferous tubules. Data are expressed as mean value±SEM (Student's *t*-test, two-tailed).

2) GFRα1-positive cell density showing the relative cell number of all A_single_∼A_aligned_ subpopulations (upper values) and A_single_ subpopulation (lower values) per mm^2^ of seminiferous tubule.

a,b In both hamsters and mice, the cell shape of GFRα1-positive (A_single_) cells is significantly (*p<0.05) more slender in the GDNF-high area than in the GDNF-low/negative area. In addition, cell processes at both the long axis and the long/short ratio (slender shape) were longer in hamsters than in mice (p<0.05).

c,d The cell number of both A_single_∼A_aligned_ spermatogonia and A_single_ spermatogonia is significantly (*p<0.05 or **p<0.01) higher in mice than in hamsters.

## Results

### A Spatiotemporal Pattern of Immunoreactive GDNF Distribution in the Seminiferous Tubules of Normal Wild-type Testes and SSC-transplanted W/W^v^ Testes in Adult Mice

**Figure 1:**
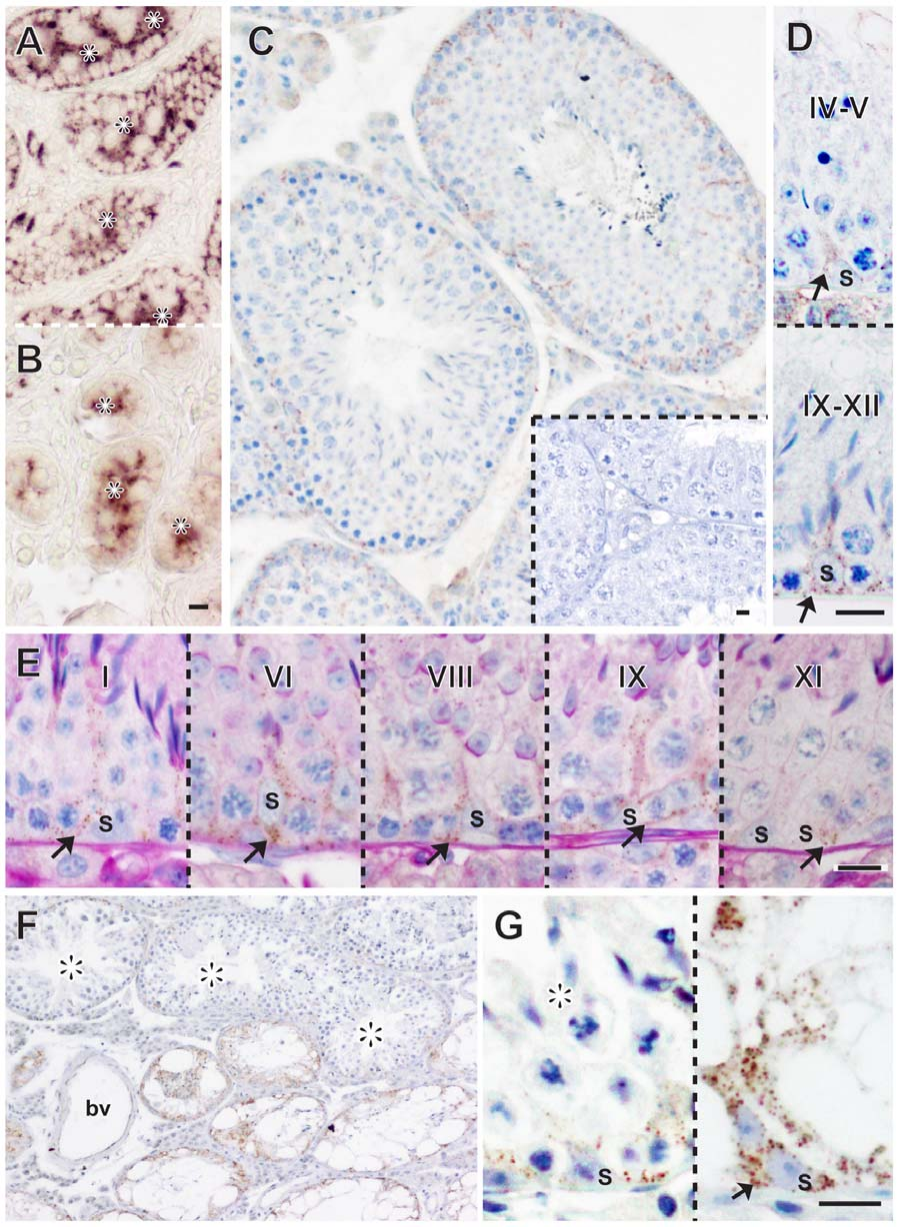
Immunoreactive GDNF expression in mouse Sertoli cells in wildtype, *Gdnf-null* and SSC-transplanted *W/W^v^* testes. (**A**, **B**) Anti-GDNF staining of wildtype (A) and *Gdnf*-null (B) testes (newborn) showing no immunoreactive GDNF signals in the basal region of the seminiferous epithelium in *Gdnf*-null testes, albeit some non-specific signals in the lumen (asterisks). (**C**–**E**) Anti-GDNF staining (brown) of wildtype adult testes showing very weak and ubiquitous GDNF expression in Sertoli cells (arrow). Plate C includes the inset plate showing a negative control section using the primary antibody pre-incubated with GDNF peptides. Plate E shows anti-GDNF (brown) and PAS (red)-double-stained images of the wildtype adult testes. (**F**, **G**) Anti-GDNF staining of the *W/W^v^* adult testes at 3 months after SSCs transplantation. The level of intensity of GDNF-positive signals is higher in the seminiferous epithelia lacking germ cells (right in G), as compared with that in the seminiferous epithelia supporting advanced germ cells (asterisks in F; left in G). Roman numerals indicate the seminiferous epithelial stage of each region. bv, blood vessel; S, Sertoli cells. Scale bars represent 10 µm.

**Figure 2:**
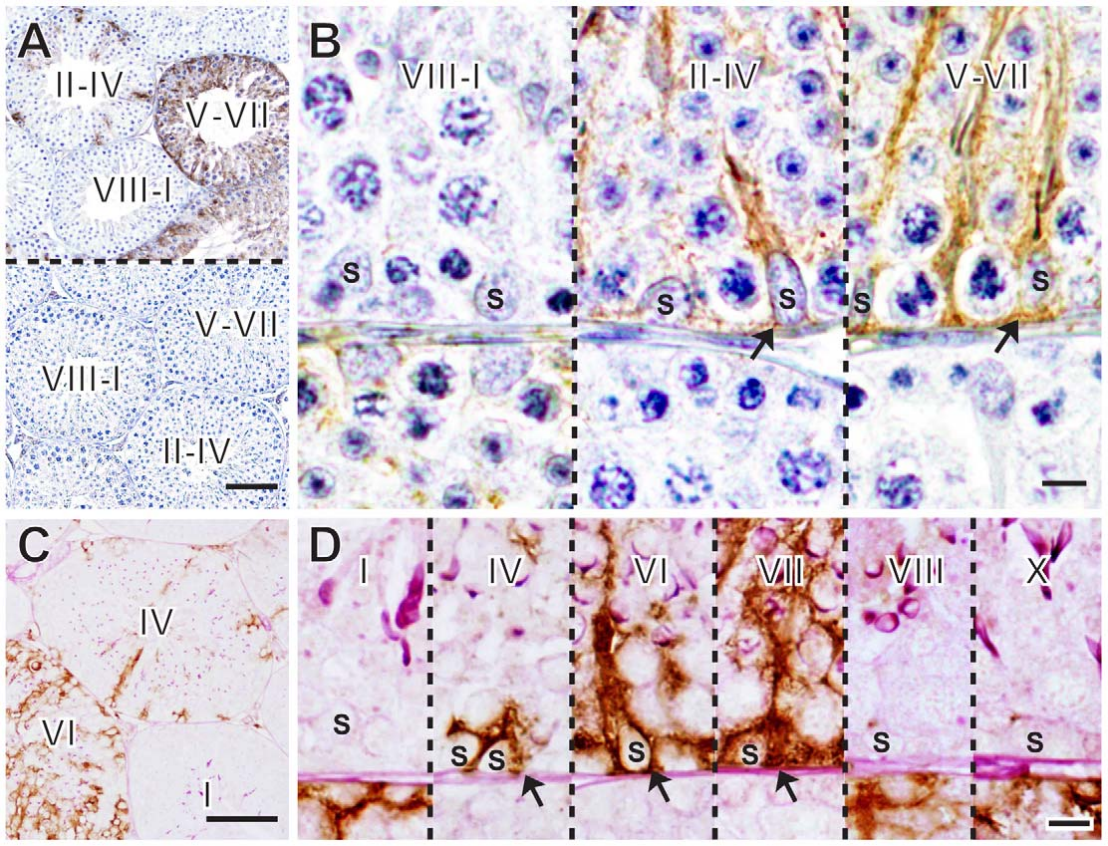
Seminiferous cycle-dependent pattern of immunoreactive GDNF expression in seminiferous tubules of “active” testes in hamsters. (**A**, **B**) Anti-GDNF staining of adult hamster testes in an “active” state showing dynamic changes in immunoreactive GDNF expression in Sertoli cells in a seminiferous cycle-dependent pattern. Negative control sections using the primary antibody pre-incubated with GDNF peptides are also shown in the lower plate of Figure A. (**C**, **D**) Anti-GDNF (brown) and PAS (red)-double-staining of the wildtype adult testes, showing that immunoreactive GDNF expression starts to occur at stages II–IV, reaches a peak at VII (arrows), and then rapidly disappears at stage VIII. Roman numerals indicate the seminiferous epithelial stage of each region. S, Sertoli cells. Scale bars in A, C represent 100 µm, while the other bars represent 10 µm.

First, GDNF expression in mouse and hamster testes was examined by conventional section immunohistochemistry (Figs. 1, 2). Of the various commercially available antibodies, we used rabbit anti-GDNF antibody against the C-terminal sequences within amino acids 155–205 of human GDNF (protein accession P39905). We confirmed the presence of highly conserved amino acid sequences corresponding to the C-terminal region of hamster GDNF with those in human, mouse and rat GDNF, and the trans-cross reactivity of this antibody to recombinant GDNF among these species (see Fig. S1). Moreover, anti-GDNF positive signals were mostly cytoplasmic and observed specifically in Sertoli cells, a major population of GDNF-secreting cells [3], [11], [13] which is located along the basal compartment of seminiferous tubules in the newborn testes (Fig. 1A). In contrast, signals were greatly reduced in the basal compartment of the seminiferous epithelia of the *Gdnf*-null newborn testes [31] (Fig. 1B). These findings suggest that immunoreactive GDNF signals are detectable by this antibody in mouse testes, although non-specific background staining was found on the luminal side.

In contrast to the high levels of GDNF expression observed in newborn testes, only weak signals were seen in Sertoli cells of the adult mouse testes (8-week-old) (Fig. 1C, 1D). Anti-GDNF and PAS (periodic acid Schiff) double-staining revealed that GDNF signals were weakly, but ubiquitously, detected in the Sertoli cells at all stages of the seminiferous epithelial cycles (Fig. 1E). This is clearly in contrast to high cyclical patterns of GDNF expression in hamster testes as described below (see Fig. 2).

In order to examine the influence of advanced spermatogenic cells on GDNF expression [29], [39], we transplanted spermatogonial stem cells (SSCs) into the *W/W^v^* testes (germ cell-less mutant due to germ cell autonomous defects) [40] and, at 3-month post-transplant, examined immunoreactive GDNF expression in the SSC-transplanted testes (Fig. 1F, 1G). In the SSC transplantation experiment, the seminiferous tubules containing donor advanced germ cells and those lacking germ cells are located close to each other within the same testis (Fig. 1F). This allows us to directly evaluate and compare the signal intensities of GDNF expression between the seminiferous epithelia supporting advanced germ cells and those lacking them within one focus area, excluding other influences in physiological and experimental conditions (e.g., interstitial environment, fixation, and all immunostaining procedures). Anti-GDNF staining showed high GDNF immunoreactivity in Sertoli cells of seminiferous tubules which lacked spermatogonia (Fig. 1F, 1G). In contrast, in seminiferous tubules which contained spermatogenic cells, the level of immunoreactive GDNF expression was low (asterisks in Fig. 1F, 1G) and similar to that in normal adult testes (Fig. 1C, 1D). These findings are in agreement with the previous data indicating higher levels of *Gdnf* mRNA expression in *W/W^v^* testes [29].

### A Spatiotemporal Pattern of GDNF Expression in the Seminiferous Tubules of Normal “Active” Testes and Short Photoperiod/Low Ambient Temperature-induced “Inactive” Testes in Adult Hamsters

Next, immunoreactive GDNF expression in adult hamster testes (8-week-old) in the “active” state was examined. Interestingly, in contrast to the less-cyclical pattern of GDNF expression in mouse testes, dynamic cyclical changes in GDNF expression were observed in normal “active” testes of hamsters (Fig. 2A, 2B; also see “Cont” in Table S1). Briefly, in 50% of seminiferous tubules, nearly all Sertoli cells showed no signals. However, in 20.5% of tubules, some Sertoli cells were found to have strong positive signals in their cytoplasm while, in 29.5% of the tubules, almost all Sertoli cells were positive for anti-GDNF staining (Fig. 2A, 2B). Anti-GDNF and PAS double-staining revealed that no signals were detected in the seminiferous tubules at stages VIII-I (Fig. 2C, 2D). At stages II–IV, some Sertoli cells located within the same cross section were positive for anti-GDNF staining, and almost all Sertoli cells were positive at stages V–VII (Fig. 2C, 2D). Interestingly, at stage VIII when spermiation (i.e., a release of matured spermatozoa from the apical area of Sertoli cells into the lumen) occurs, a rapid reduction in GDNF-positive signals was observed in all Sertoli cells, leading to the loss of GDNF expression during stages VIII-I. This immunostaining pattern is clearly in agreement with the present in situ hybridization data showing dynamic cyclical patterns of *Gdnf* expression at high levels before spermiation stages (see Fig. S2).

**Figure 3:**
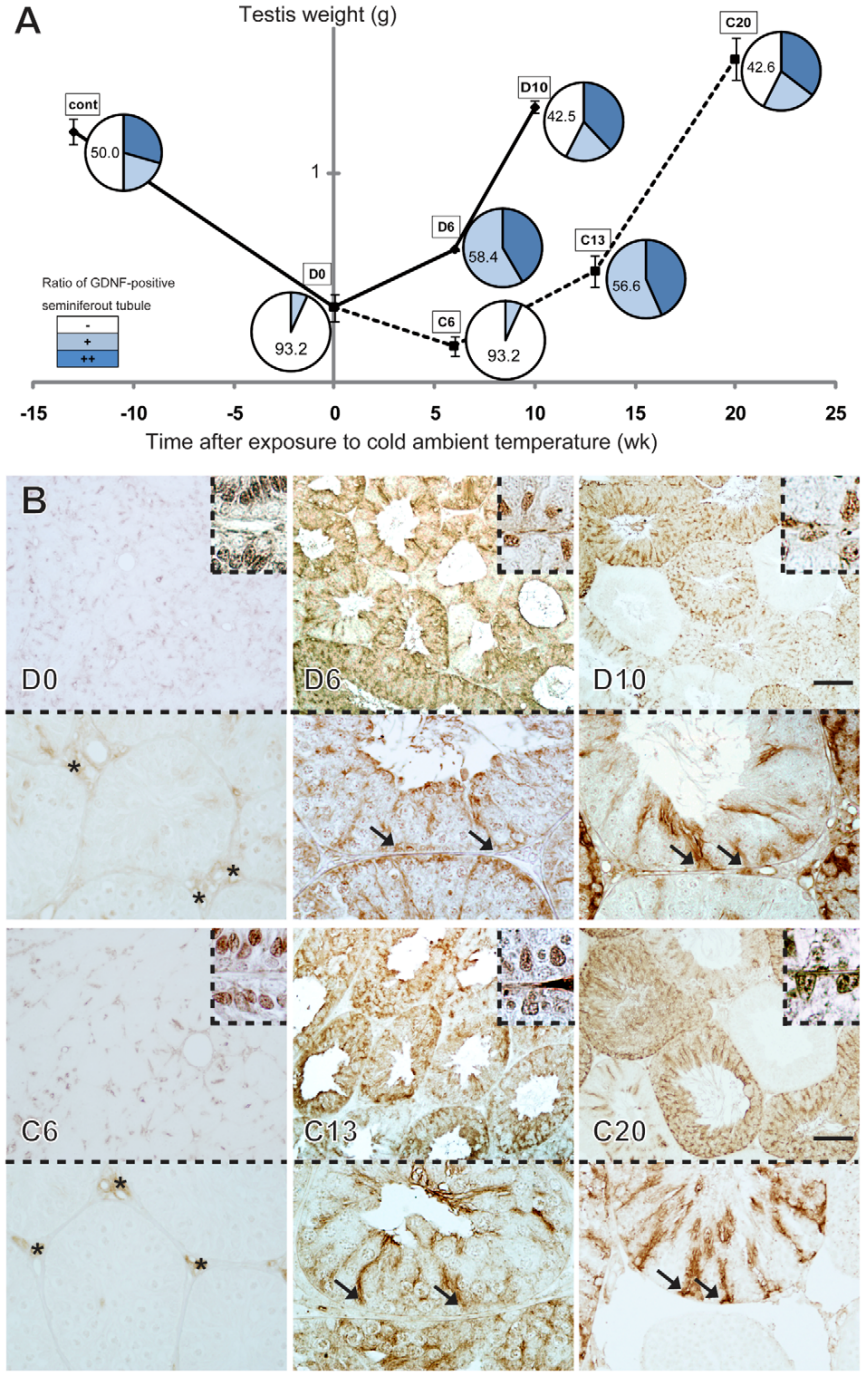
Changes in immunoreactive GDNF expression in hamster testes in photoregression, hibernating and subsequent recrudescent states. Adult hamsters (8-week-old) were exposed to a short photoperiod (6 h light, 18 h dark) and an ambient temperature of 23°C. The testes photoregressed to the most “inactive” state at 13 weeks of treatment (D0). Then, in half of the hamsters, the ambient temperature was reduced from 23°C to 5°C to induce a hibernated state (C6). It was shown that, after 13 weeks of exposure to a short photoperiod (D0), spermatogenic activity spontaneously recrudesced after 10 to 20 weeks in both the 5°C and 23°C groups (“D10” and “C20”), albeit a 7 to 10 week delay in GDNF expression was observed in the 5°C group. (**A**) A line graph, including small circle graphs at each stage, shows changes in testicular weight (Y axis in the line graph; error bars indicate±SD) and the appearance ratio of GDNF-positive seminiferous tubules (small circle graph at each stage; also see Table S1) in adult testes exposed to a short photoperiod (solid lines in the line graph; D0, D6, and D10) in combination with a low ambient temperature (broken lines in the line graph; C6, C13, and C20). X axis represents weeks before and after the most “inactive” state (D0) was reached in Week 13 of treatment. (**B**) Anti-GDNF immunostaining patterns show no appreciable positive signals in almost any of the seminiferous tubules in “inactive” testes at D0 and C6 stages. A rapid recovery of immunoreactive GDNF-positive signals is ubiquitously observed, even at C13 stage (arrows) when the testicular weight is at a similarly low level to that in the inactive state, D0 (“D0”, “C13” in A, B). A seminiferous cycle-dependent pattern of GDNF signals recovers at D10 and C20. The insets in B indicate the constant GATA4 expression in the Sertoli cells throughout all stages (insets in B). Asterisks, non-specific signals in interstitial region. Scale bars represent 100 µm.

It is well known that *Gdnf* expression in Sertoli cells is positively up-regulated by FSH [29], [30]. In seasonally breeding hamsters, it has been shown that a short photoperiod and a low ambient temperature can induce the most “inactive” state of spermatogenic activity with low levels of serum FSH/LH [41], [42]. In order to confirm reduced GDNF expression in Sertoli cells in an “inactive” state, we examined immunoreactive GDNF expression in the hamster testes in the photoregressed and hibernating states and during subsequent spontaneous recrudescence by prolonged exposure to inhibitory photoperiods (Fig. 3, see Fig. S3).

In adult hamsters (8-week-old) that were exposed to a short photoperiod (6 h light, 18 h dark) and an ambient temperature of 23°C, the testes photoregress to the most “inactive” state at Week 13 of treatment, showing atrophied seminiferous tubules with closed lumen [32]. As anticipated, anti-GDNF staining showed a marked reduction in GDNF-positive signals in the inactive testes at Week 13 (“D0” in Fig. 3A, 3B, also see Table S1), suggesting the down-regulation of GDNF expression in the gonadally inactive stages. When the hamsters were transferred from an ambient temperature of 23°C to 5°C at Week 13 and maintained in a short photoperiod (5°C group), most animals entered a hibernated state within 4 to 8 weeks of transfer. At this stage, no appreciable signals were detected in almost any of the seminiferous tubules in these testes (“C6” in Fig. 3A, 3B). This finding is in contrast to the constantly moderate levels of nuclear-positive signals for anti-GATA4 staining in Sertoli cells throughout all (i.e., inactive and active) stages examined in this study (inset of Fig. 3B). After exposure to a short photoperiod for 13 weeks, spermatogenic activity began to spontaneously recrudesce with complete recovery observed within 6 to 10 weeks in the 23°C group (“D6” and “D10” in Fig. 3A, 3B, respectively) or within 13 to 20 weeks in the 5°C group (“C13” and “C20” in Fig. 3A, 3B, respectively). GDNF expression in the 5°C group, however, was delayed by 7 to 10 weeks. As anticipated, anti-GDNF staining showed a rapid recovery of GDNF-positive signals even in D6 and C13 before the recovery in testicular weight to the active level (“D6”, “C13” in Fig. 3A, 3B). This finding is consistent with previous reports showing a rapid recovery in plasma FSH/LH levels within 1–3 weeks prior to recrudescence of spermatogenic activity in adult photoinhibited hamster [42]–[44].

### Whole-mount Immunostaining Visualized Cyclical and Patch-like GDNF-positive Deposits along the Basal Surface of Sertoli Cells in Hamster and Mouse Testes

**Figure 4:**
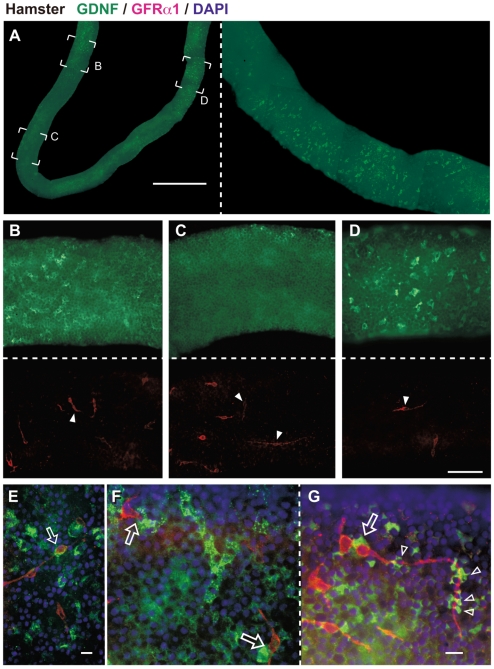
Cyclical changes in immunoreactive GDNF deposits and their close co-localization with GFRα1-positive spermatogonia in hamsters. (**A**–**D**) Whole-mount immunostaining (without permeabilization) of seminiferous tubules showing GDNF-positive deposits (green) and GFRα1-positive spermatogonia (red) in the basal compartment of the seminiferous epithelia. In panel A, the right inset plate indicates a moderately magnified image of the right-half area of the left plate, showing a striatal distribution of the patch-like GDNF-positive signals in the border region between GDNF-negative to -high area. Panels B-D are higher magnification images of the tubule of panel A (B, the border region between GDNF-high and -low/negative areas; C, GDNF-low/negative area; D, GDNF-high area). Arrowheads indicate GFRα1-positive spermatogonia with long slender cell processes. (**E**–**G**) Higher magnified images (green, GDNF; red, GFRα1; blue, DAPI) show the close co-localization of GDNF deposits and a subpopulation of GFRα1-positive spermatogonia. The long cell processes (arrowheads) or cell bodies (open arrows) are closely associated with some GDNF-positive deposits. Scale bars represent 1 mm in A, 100 µm in D, and 10 µm in E, G.

**Figure 5:**
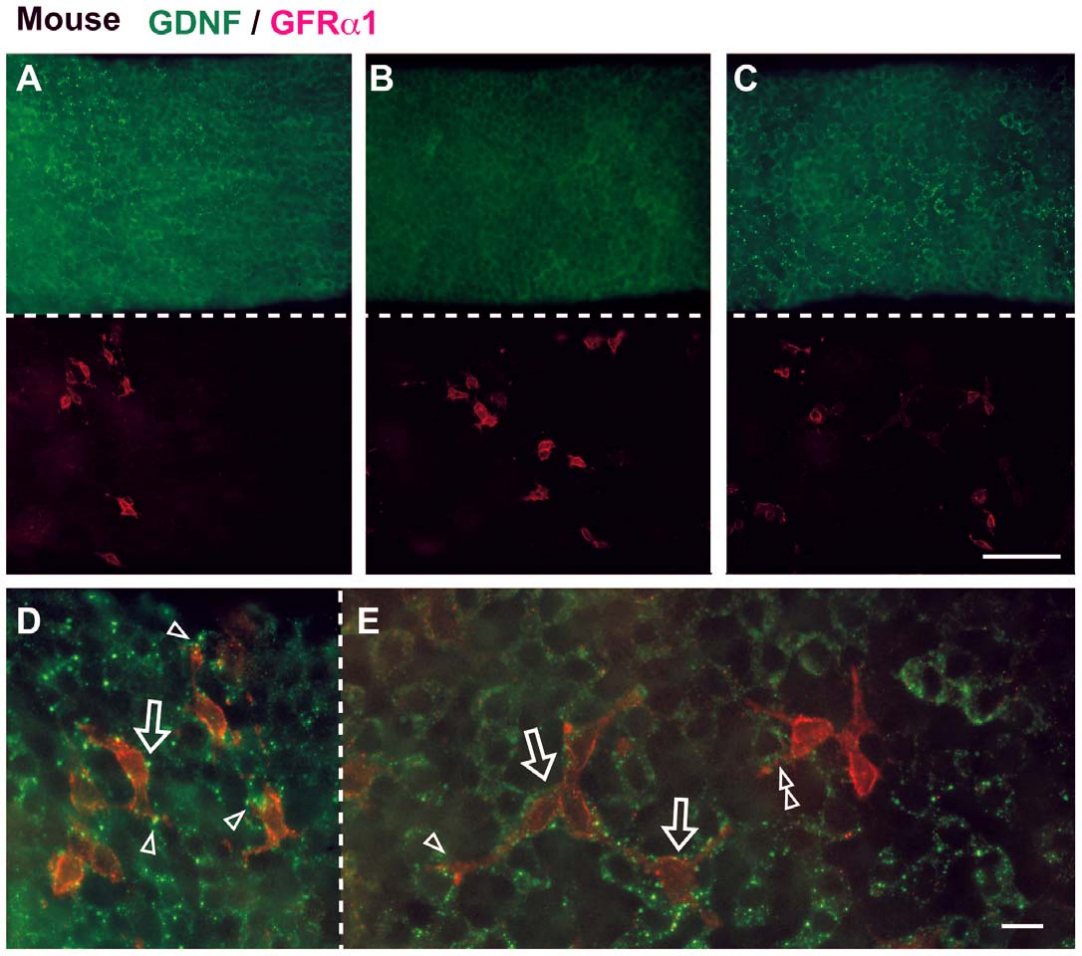
Immunoreactive GDNF deposits and their close co-localization with a subpopulation of GFRα1-positive spermatogonia in mice. Whole-mount immunostaining (without permeabilization) of seminiferous tubules showing GDNF-positive deposits (green) and GFRα1-positive spermatogonia (red) in the basal surface of seminiferous epithelia in mice. (**A**–**C**) Plate A shows the border region between GDNF-high (left side) and -low/negative (right side) surface areas (Note the tilted distribution of GFRα1-positive cells on the left side). Plates B and C show GDNF-low/negative and -high tubule areas, respectively. (**D**, **E**) Merged images visualize the close co-localization of granular GDNF deposits and some GFRα1-positive spermatogonia. Plates D and E are higher magnification images of the tubule of panels A and C, respectively. The patch-like distribution of GDNF-positive granular deposits is closely associated with the outline of a subpopulation of spermatogonia with a close connection with the cell processes (arrowheads) and cell bodies (open arrows). A double arrowhead indicates the asymmetrical co-localization of GDNF deposits and connected cells of A_paired_ GFRα1-positive cells (Note the lack of GDNF deposits around another A_paired_ GFRα1-positive cell). Scale bars represent 100 µm in C and 10 µm in E.

As described above, anti-GDNF section immunohistochemistry showed species-specific as well as seminiferous cycle- and spermatogenic activity-dependent changes in immunoreactive GDNF expression in Sertoli cells. It is unclear, however, which GDNF-positive signal sites correspond to the extracellular GDNF molecules that can be accessed by GFRα1-positive spermatogonia in vivo. Therefore, in order to selectively visualize GDNF molecules in the extracellular region of the basal compartment of seminiferous epithelia, we applied anti-GDNF staining for PFA-fixed whole seminiferous tubules without any permeabilization steps in the adult testes of hamsters (Fig. 4) and mice (Fig. 5). Whole-mount anti-GDNF immunostaining showed seminiferous cycle-dependent patterns in immunoreactive granular GDNF signals along the basal wall of the seminiferous tubules in hamsters, albeit with slightly cyclical patterns in mice (green fluorescence in Figs. 4A–D, 5A–C; also see negative controls in Fig. S4). Fluorescent microscopy of DAPI images of round and elongate spermatids showed that the border between immunoreactive GDNF-high and GDNF-negative areas roughly corresponded to the stages VII/VIII, just at spermiation in hamsters (Fig. 2; data not shown). In mice, the GDNF-high and –low/negative regions were also distinguishable along the basal wall of the seminiferous tubules (Fig. 5A–C), although such cyclical patterns were not evident in the immunostained sections (Fig. 1C–E). Interestingly, whole-mount immunostaining visualized a patch-like distribution pattern of granular GDNF deposits along the basal wall of the seminiferous tubules in hamsters (green in Fig. 4B, 4D–G). Even in the GDNF-positive area during the peak of its expression, GDNF deposits appeared to be restricted to a regionally defined, patch-like distribution pattern along the basal surface areas of Sertoli cells (Fig. 4F). This is in sharp contrast to almost all Sertoli cells becoming positive for anti-GDNF staining in transverse sections in hamsters (“V–VII” in Fig. 2). In mice, GDNF-positive deposits were more granular in shape and wider in distribution than those in hamsters (green in Fig. 5A–E). In some regions, these granular deposits appeared to be, at times, defined by a patch-like restricted area similar to the GDNF-positive patches seen in hamsters (green signals in Fig. 5E).

**Figure 6:**
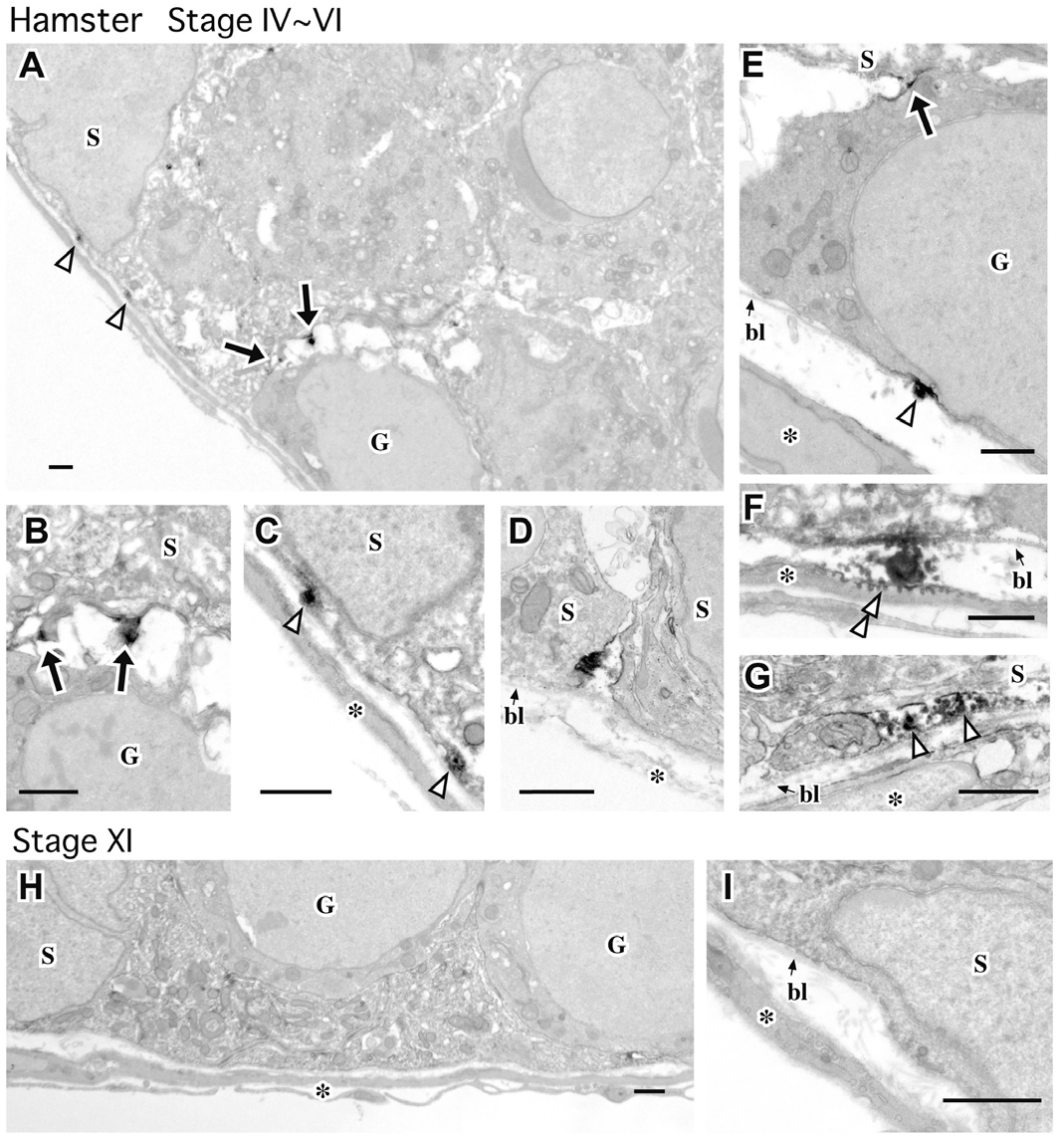
Immunoelectron microscopic analysis showing GDNF-positive signals along the basal surface of Sertoli cells in hamsters. Electron micrographs showing transverse ultrathin sections of whole seminiferous tubules immunostained with anti-GDNF antibodies (DAB-osmium black). GDNF-positive signals are observed in the basal surface of Sertoli cells (S) adjacent to the spermatogonia (arrows) and the basal lamina (open arrowheads). Some signals are detected in the pinocytotic vesicles of peritubular myoid cells (double arrowhead in F). No signals are detected in the basal compartment of seminiferous tubules at stage XI (H, I). Panels B and C are higher magnified images of the basal surface of Sertoli cells in panel A. Asterisks, peritubular myoid cells; bl, basal lamina; G, spermatogenic cells; S, Sertoli cells. Scale bars represent 1 µm.

Immunoelectron microscopy of whole-mount samples stained with the same non-permeabilization procedures revealed that the majority of GDNF-positive signals were located within the extracellular space adjacent to the spermatogonia and basal lamina along the basal surface of some Sertoli cells (arrows and arrowheads in Fig. 6). Moreover, certain weak signals were detectable in the outer tubular region between the basal lamina and peritubular myoid cells and in the transported vesicles in the cytoplasm of the peritubular myoid cells (double-arrowhead in Fig. 6F), suggesting a possible removal process of the GDNF molecules from the basal compartment of the seminiferous epithelium through the basal lamina and peritubular myoid cells. Taken together, these data suggest that patch-like GDNF deposits are formed in a seminiferous cycle-dependent manner in the extracellular space along the basal surface of Sertoli cells. The present non-permeabilized condition of whole-mount staining allowed us to mainly detect GDNF-positive signals within the extracellular spaces of the basal compartment in seminiferous tubules. However, we should consider the presence of cytoplasmic GDNF-positive signals near the plasma membrane of the Sertoli cells as a possible source of some GDNF-positive signals in this whole-mount staining.

### Close Co-localization of Immunoreactive GDNF Deposits with a Subpopulation of GFRα1-positive Spermatogonia in the Basal Compartment of Seminiferous Tubules in both Hamster and Mouse Testes

Finally, we visualized a possible interaction between immunoreactive GDNF molecules and its GPI-linked cell surface receptor, GFRα1, on undifferentiated spermatogonia including SSCs, by whole-mount double-staining of seminiferous tubules (without permeabilization). GFRα1/GDNF-double-staining allowed us to quantitatively compare the number and shape of GFRα1-positive cells (A_single_∼A_aligned_) in GDNF-high and -low/negative areas of seminiferous tubules (Table 1). In both hamster and mouse seminiferous tubules, the relative number of GFRα1-positive spermatogonia tended to be increased in the GDNF-high area as compared with that in the GDNF-low/negative area (Table 1). Moreover, we occasionally noticed a tilted distribution of GFRα1-positive cells toward the GDNF-high area in the border region between GDNF-high and -low/negative areas (see Fig. 5A; GDNF-high left-half area versus GDNF-low/negative right-half area). As for cell morphology, GFRα1-positive cells (A_single_) in the GDNF-high area were significantly (p<0.05) more slender in shape, as compared with cells in the GDNF-low/negative area in both mice and hamsters (Table 1). As for species differences, GFRα1-positive cells were significantly more slender in shape and lower in number in hamsters, as compared with GFRα1-positive cells in mice (Table 1; see Fig. S5).

**Figure 7:**
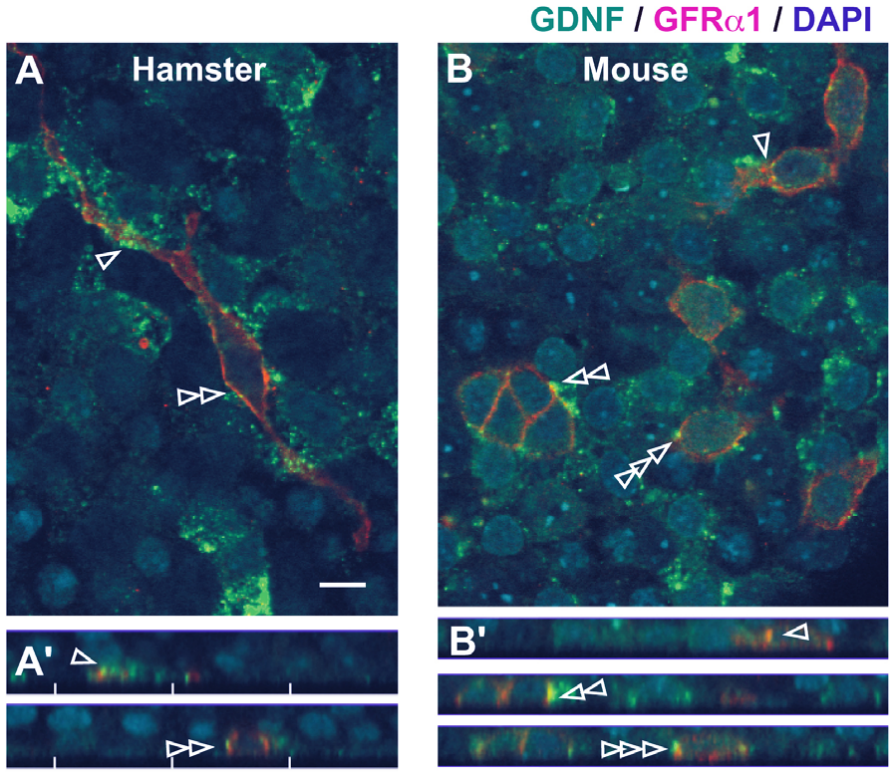
Co-localization of GDNF-positive signals with the apical surface of GFRα1-positive spermatogonia in hamsters and mice. The seminiferous tubules were stained with anti-GDNF (green) and GFRα1 (red) antibodies (DAPI, blue), and then analyzed to reconstruct a three-dimensional image (see also Movie S1 and Movie S2). GDNF deposits are closely connected to the cell processes and cell bodies (open arrowheads) of some GFRα1-positive spermatogonia in hamsters (A) and mice (B). Plates A and B indicate X-Y axis sections, while plates A′ and B′ represent reconstructed X-Z axis sectioning images at the points indicated by each arrowhead. Scale bar represents 10 µm.

Interestingly, GFRα1/GDNF double-staining visualized the close co-localization of GDNF deposits and a subpopulation of GFRα1-positive cells in the basal region of the seminiferous tubules in both hamster and mouse (open arrows in Figs. 4, 5). The long cell processes of some GFRα1-positive cells were closely connected with the patch-like distribution of immunoreactive GDNF-positive deposits in hamster seminiferous tubules (arrowheads in Fig. 4G). In some A_paired_ and A_aligned_ subpopulations of GFRα1-positive cells, asymmetrical co-localization with GDNF deposits was observed among connected GFRα1-positive cells (e.g. “open arrow” in Fig. 4E; double arrowheads in Fig. 5E). Confocal microscopy clearly revealed that the apical surface of some GFRα1-positive cells was colocalized to GDNF-positive signals, suggesting a possible interaction site between GDNF and GFRα1-positive cells in the basal compartment of seminiferous tubules (Fig. 7; see Movie S1, Movie S2).

**Figure 8:**
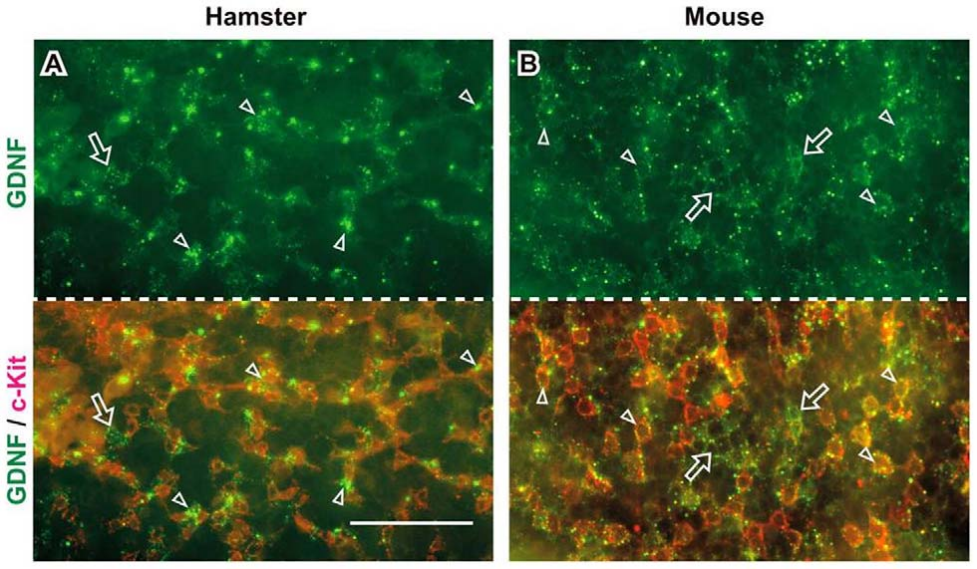
GDNF-positive signals largely overlap with the areas of c-kit-positive spermatogonia in hamsters and mice. Whole-mount immunostaining (without permeabilization) of seminiferous tubules showing GDNF-positive deposits (green) and c-kit-positive spermatogonia (red) in the basal surface of seminiferous epithelia in hamsters (A) and mice (B). Merged images (lower plates) visualize that the majority of GDNF deposits are overlapped with the areas of the c-kit-positive differentiated spermatogonia (arrowheads). Arrows indicate GDNF deposits that are seen in the c-kit-negative areas. Scale bar represents 100 µm.

On the other hand, we also noticed that a considerable number of GFRα1-positive cells were not directly associated with any GDNF deposit, especially in hamsters (see Figs. 4C, 5B). Both in mice and hamsters, the majority of GDNF-positive signals appear to correspond to the cell surface area of the c-kit-positive differentiated spermatogonia (Fig. 8) rather than the cell process or cell body of GFRα1-positive cells in the basal compartment (Fig. 4E–G; Fig. 5D,E).

## Discussion

This study was the first to visualize the changes in immunoreactive GDNF expression in the adult testes in a species-specific as well as spermatogenic activity- and seminiferous cycle-dependent manner. As anticipated, GDNF expression was specifically observed in Sertoli cells of the seminiferous epithelia, and its expression levels appear to be tightly regulated by the spermatogenic activity of the testes in both mice and hamsters. In mice, higher levels of GDNF expression were observed in seminiferous epithelium lacking germ cells than in seminiferous tubules colonized by donor germ cells (Fig. 1F, 1G), which may reflect a positive response to compensate for the reduced germ cell number in the basal compartment. In contrast, lower levels of GDNF expression were noted in almost all Sertoli cells in photoregressed and hibernating hamster testes (Fig. 3). Moreover, during the subsequent testicular recrudescence, GDNF expression was shown to be clearly up-regulated at the initial phases which coincide with the resumption of spermatogenesis (Fig. 3). Since a rapid recovery in serum FSH/LH levels occurs before testicular function in the adult photoinhibited hamster [42], [44], this is clearly consistent with the previous data that GDNF expression is tightly regulated immediately downstream of the gonadotropin-gonadal axis [29], [30]. Moreover, in “active” testes in hamsters, the stages II–VII of the high levels of GDNF-positive signals in the Sertoli cells (Fig. 2C, 2D) roughly coincide with those of the highest levels of FSH-induced cAMP production in the seminiferous epithelia (stages II–VI) [45], [46].

Anti-GDNF immunostaining showed high levels of immunoreactive GDNF expression at the timing of spermiation in a seminiferous cycle-dependent pattern in hamsters. It is well known that, at the same seminiferous cycle stage as spermiation (stages VII/VIII in hamsters or stages VIII/IX in mice), preleptotene spermatocytes move across the blood-testis barrier from the basal to the adluminal compartment of the seminiferous epithelium [47], [48]. It is likely that the transition of preleptotene spermatocytes from the basal to adluminal compartment also leads to a transient increase in “spare room” for spermatogonia within the basal compartment of the seminiferous epithelium. Since higher levels of GDNF expression were observed in seminiferous epithelia lacking germ cells than in seminiferous epithelia colonized by germ cells in SSC-transplanted *W/W^v^* testes, these data suggest that some other signals which are derived from the presence or absence of “spare room” and/or advanced spermatogenic cells within the basal compartment may partially contribute to cyclical changes in GDNF expression in mammalian spermatogenesis.

The present anti-GDNF staining of whole seminiferous tubules successfully visualized the cyclical and patch-like distribution patterns of GDNF-positive granular deposits along the basal surface of Sertoli cells in both species. Double-staining of GDNF and its receptor, GFRα1, showed close co-localization of GDNF deposits and a subpopulation of GFRα1-positive spermatogonia in the basal region. Moreover, the present quantitative analysis revealed that GFRα1-positive cells showed a slender bipolar shape as well as a tendency for increased cell numbers in the GDNF-enriched area, as compared with those in the GDNF-low/negative area of the seminiferous tubules. For morphometric determination, further studies are required to generate hard data which can be statistically verified by using more accurate quantification of the GDNF signal levels around each GFRα1-positive cell. However, these findings suggest that GDNF-positive deposits along the basal surface of Sertoli cells have a “niche” function in the in vivo maintenance of SSCs in mammalian testes. These GDNF-positive deposits may not be static, but unstable dependently on the functional states and seminiferous cycle stages, although this does not fit the classical niche for the SSCs as a fixed and static structure in several lower vertebrates and invertebrate species [49]–[51].

The cyclical and patch-like distribution of GDNF deposits along the basal surface of Sertoli cells possibly leads to the asymmetric interaction of GDNF signals with some A_paired_ and A_aligned_ GFRα1-positive cells (arrow in Fig. 4E; double-arrowhead in Fig. 5E), which may be consistent with recent suggestion showing asymmetric selection of SSCs from A_aligned_ spermatogonia after fragmentation in vivo [7]. Moreover, the present regionalized GDNF regulation in a small subpopulation of GFRα1-positive cells would explain the findings of a recent clone-fate study which showed that SSCs have an unexpectedly short life-span (average: ≤2 weeks) in the seminiferous epithelia [52]. This is because, in both hamster and mouse, many GFRα1-positive cells do not appear to physically associate and co-localize with GDNF deposits (see Figs. 4C, 5B), possibly leading to their eventual removal from a potential SSCs pool. Taken together, it is reasonable to speculate that such regionalized GDNF regulation may define the size of a pool of GFRα1-positive spermatogonia, especially in hamsters, possibly leading to the finely-tuned control between the self-renewal/survival and differentiation of the SSCs in the basal compartment of seminiferous tubules. Moreover, the present study demonstrated that the GDNF-positive signals are accumulated largely on the c-kit-positive spermatogonia along the basal surface of the seminiferous tubules (Fig. 8). This in turn suggests that the dynamics of a c-kit-positive population of A_aligned_ spermatogonia clearly affects the size of a pool of GFRα1-positive spermatogonia (mostly A_single_ and A_paired_) in a positive feedback fashion. The components of GDNF-positive granular deposits, their association with the blood vessels, interstitial cells, peritubular myoid cells, and the molecular mechanisms underlying their distribution and turnover within the basal compartment of seminiferous tubules, could be a focus for future studies.

Kanatsu-Shinohara et al. (2008) [22] reported that the general characteristics of hamster germline stem (GS) cells are similar to those of mouse and rat GS cells, indicating a conserved GDNF action of self-renewal and maintenance of the SSCs pool between seasonal and non-seasonal breeding rodents [53]. Interestingly, we noticed the following species-specific differences in the expression profiles of GDNF and GFRα1 between mouse and hamster testes: 1) Hamster GFRα1-positive spermatogonia are more slender in shape and lower in cell density than those in mice, 2) GDNF expression in hamsters is more cyclical, is restricted to a narrower area along the longitudinal seminiferous tubule (i.e., only at stages II∼VII), and consists of patch-like deposits. In contrast, GNDF expression in mice is ubiquitous/less cyclical, with granular GDNF deposits in a wider area along the longitudinal seminiferous tubule. These findings imply that the less cyclical and ubiquitous GDNF distribution in mice is closely associated with the maintenance of a large number of GFRα1-positive cells. On the other hand, the more restricted GDNF distribution would explain the relatively small number of GFRα1-positive spermatogonia in hamsters, as compared with that in mice. Interestingly, hamster GFRα1-positive spermatogonia are significantly more slender in shape than those in mice, which might possibly reflect the high migratory activity in hamsters. This small number of GFRα1-positive cells with a high migratory activity may have advantages over a SSCs pool which is rapidly changing in size during the transition between inactive and active states in seasonal breeding animals. This is because the up- and down-regulation of GDNF expression is directly transmitted to the rapid expansion of, and/or reduction in, the SSCs pool throughout the longitudinal seminiferous tubule. This observation is consistent with the present data which demonstrated the ubiquitous and widespread nature of GDNF expression in most seminiferous tubules in the initial phases of spontaneous testicular recrudescence in hamsters (“D6”, “C13” in Fig. 3). Both GDNF and GFRα1 may be highly conserved molecules between mice and hamsters [22], reflecting the successful maintenance and colonization of hamster SSCs in mouse testicular soma [53] and the higher cross-species reactivity of anti-GDNF and anti-GFRα1 antibodies (this study). Taken together, these findings indicate that the hamster testes in photoregressed, hibernating and subsequent recrudescent states are very useful in a comparative animal approach to understand the seasonal regulation and evolution of the SSCs and their niche in mammalian spermatogenesis.

In conclusion, the present study was the first to demonstrate the dynamic changes in immunoreactive GDNF expression and its close association with a small subpopulation of GFRα1-positive spermatogonia in the basal compartment of seminiferous epithelia. The unexpectedly cyclical and patch-like distribution of GDNF deposits implicates a novel hypothesis for in vivo maintenance of SSCs based on highly regionalized association between GFRα1-positive cells and extracellular GDNF signals in the basal compartment of the seminiferous epithelia of mammalian testes.

## Supporting Information

Figure S1 **Comparative amino acid sequences of the C-terminal region of hamster, human, mouse and rat GDNF (A) and cross-reactivity of anti-human GDNF antibody with rat recombinant GDNF proteins by SDS-PAGE/western blot analysis.** (**A**) The C-terminal amino acid sequences of GDNF (the epitope region of anti-GDNF antibody used in this study [against the amino acids 155–205 of human GDNF: acc no. P39905], arrows in A) are highly conserved among human [P39905], hamster [direct sequencing of RT-PCR products; this study], mouse [P48540], and rat [Q07731] (using ClustalW multiple alignment software). (**B**) Trans-species cross-reactivity of this antibody with functionally active recombinant rat GDNF proteins (90.2% [46/51] amino acid identity with the human GDNF epitope; 15 or 30 kDa as monomer or dimmer; PeproTech). The immunoblot control by normal rabbit IgG is also shown in the right-most lane.
(TIF)

Figure S2 **Whole-mount (A–C) and section (D–E) in situ hybridization analyses showing high** ***Gdnf*** **expression before spermiation (∼ stage VII) in hamster testes.** (**A–C**) Whole-mount in situ hybridization analysis reveals seminiferous cycle-dependent expression of *Gdnf* mRNA in hamster seminiferous tubules (purple staining). In A and B, arrowheads indicate the border between high- and low *Gdnf*-positive areas. In C, SBA lectin staining (red fluorescence for acrosome staining; DAPI, blue in lower plate) using transverse sections of whole-mount stained seminiferous tubules (*Gdn*f signal, purple; upper plate) reveals the reduction in *Gdnf* expression between stages VII and VIII (inset indicates positive signals in Sertoli cells at stage VII). The changes are consistent with the immunohistochemical data (Fig. 2). (**D–E**) Section in situ hybridization analysis demonstrates high levels of *Gdnf* expression before spermiation in hamster testes (purple staining). Asterisks, non-specific signals in the acrosomes of round spermatids. Scale bars represent 100 µm in C and D, and 10 µm in E.
(TIF)

Figure S3 **Histological analysis of seminiferous tubules in short photoperiod/low ambient temperature-induced “inactive” testes in adult hamsters.** Adult hamsters (8-week-old) were exposed to a short photoperiod (6 h light, 18 h dark) and an ambient temperature of 23°C. After the testes reached the most “inactive” state in Week 13 of treatment (D0), half of the hamsters were maintained in an environment with an ambient temperature of 5°C (5°C group) for 6 (C6), 13 (C13), or 20 weeks (C20), respectively. The remaining hamsters were maintained in an environment with a stable ambient temperature of 23°C (23°C group) for 6 (D6) or 10 weeks (D10), respectively. After exposure to a short photoperiod for 13 weeks (D0), spermatogenic activity began to recover autonomously, with complete recovery observed within 10 to 20 weeks in both the 5°C (C20) and 23°C groups (D10). Scale bars represent 100 µm.
(TIF)

Figure S4 **Negative controls for whole-mount anti-GDNF immunostaining (without permeabilization) of seminiferous tubules in hamsters and mice.** Anti-GDNF antibody was pre-incubated with GDNF peptides (sc-328P; Santa Cruz Biotechnology) prior to use for whole mount immunostaining. The pre-treatment with GDNF peptides (+pep) greatly reduced GDNF-positive signals in both hamster (A, B) and mouse (C, D) samples. Each plate includes the inset panel showing a higher magnification image of upper panel. Scale bar represents 100 µm.
(TIF)

Figure S5 **Comparative GFRα1/GDNF-double-staining images of the seminiferous tubules in hamsters and mice.** Whole-mount immunostaining (without permeabilization) of seminiferous tubules showing GDNF-positive deposits (green) and GFRα1-positive spermatogonia (red) in the basal compartment of the seminiferous epithelia in hamsters (left) and mice (right). In each plate, the seminiferous tubule is shown at the same magnification. In the left plate, the lower edge of the tubule wall is missing due to the larger diameter of the seminiferous tubule in hamster than that of the mouse. Hamster GFRα1-positive cells are more slender in shape and lower in number than those in mouse GFRα1-positive cells. In both plates, dotted lines roughly indicate the border between GDNF-high and -low/negative areas of the seminiferous tubules. Scale bar represents 10 µm.
(TIF)

Movie S1 **Rotating 3D reconstruction showing the close co-localization of a GFRα1-positive spermatogonial cell with immunoreactive GDNF-positive deposits in the basal compartment of seminiferous tubule in hamster testes.** PFA-fixed seminiferous tubule fragments were double-stained with anti-GDNF (green) and GFRα1 (red) antibodies (DAPI, blue) without any permeabilization steps, and then analyzed to reconstruct a three-dimensional image using an Olympus FluoView confocal laser microscope (FV10i; Olympus, Japan) in combination with Volocity software (Mitani Sangyo, Japan) (see also Fig. 7).
(MP4)

Movie S2 **Rotating 3D reconstruction showing the close co-localization of a GFRα1-positive spermatogonial cell with immunoreactive GDNF-positive deposits in the basal compartment of seminiferous tubule in mouse testes.** PFA-fixed seminiferous tubule fragments were double-stained with anti-GDNF (green) and GFRα1 (red) antibodies (DAPI, blue) without any permeabilization steps, and then analyzed to reconstruct a three-dimensional image using an Olympus FluoView confocal laser microscope (FV10i; Olympus, Japan) in combination with Volocity software (Mitani Sangyo, Japan) (see also Fig. 7).
(MP4)

Table S1 **Ratio of GDNF-positive seminiferous tubule in hamster testes at inactive and recovery period.**
(DOC)
