# Supplementary figures and images for: Cyclical and Patch-Like GDNF Distribution along the Basal Surface of Sertoli Cells in Mouse and Hamster Testes
Source: PLoS One. 2011 Dec 9;6(12):e28367. doi: 10.1371/journal.pone.0028367 (PMC3235125; doi:10.1371/journal.pone.0028367)

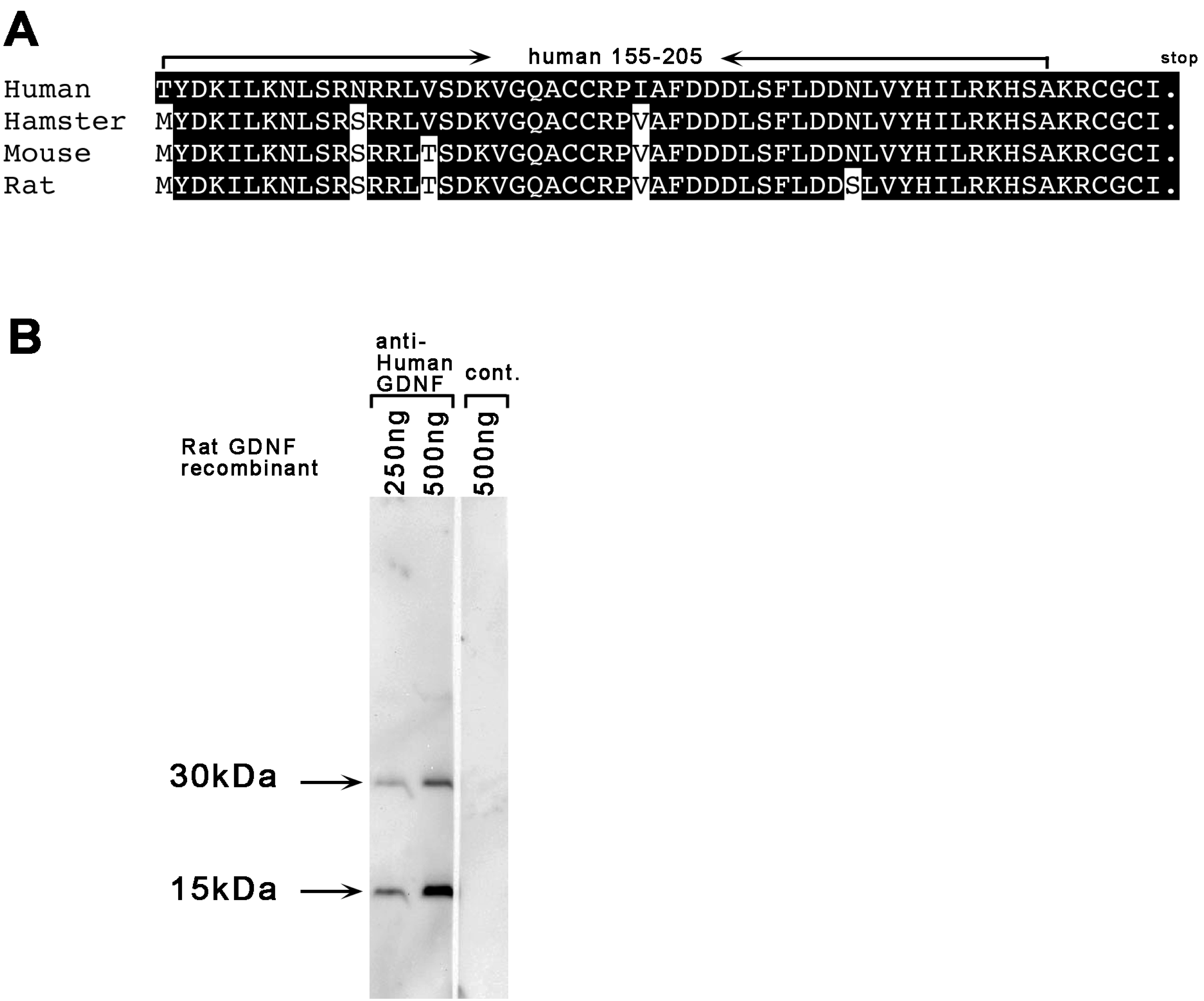

Supplement: Figure S1 — Comparative amino acid sequences of the C-terminal region of hamster, human, mouse and rat GDNF (A) and cross-reactivity of anti-human GDNF antibody with rat recombinant GDNF proteins by SDS-PAGE/western blot analysis. (A) The C-terminal amino acid sequences of GDNF (the epitope region of anti-GDNF antibody used in this study [against the amino acids 155–205 of human GDNF: acc no. P39905], arrows in A) are highly conserved among human [P39905], hamster [direct sequencing of RT-PCR products; this study], mouse [P48540], and rat [Q07731] (using ClustalW multiple alignment software). (B) Trans-species cross-reactivity of this antibody with functionally active recombinant rat GDNF proteins (90.2% [46/51] amino acid identity with the human GDNF epitope; 15 or 30 kDa as monomer or dimmer; PeproTech). The immunoblot control by normal rabbit IgG is also shown in the right-most lane. (TIF) [file pone.0028367.s001.tif]

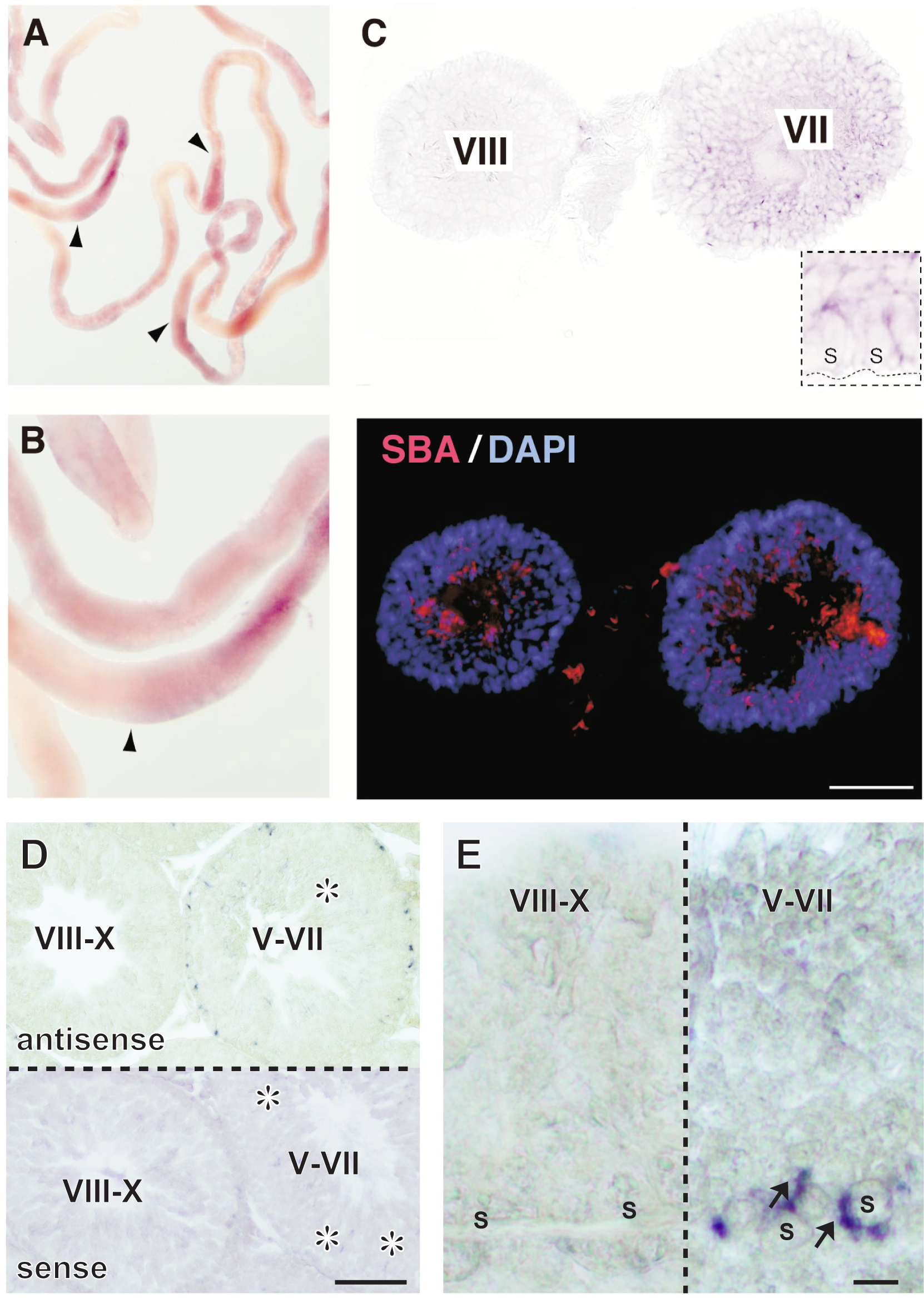

Supplement: Figure S2 — Whole-mount (A–C) and section (D–E) in situ hybridization analyses showing high Gdnf expression before spermiation (∼ stage VII) in hamster testes. (A–C) Whole-mount in situ hybridization analysis reveals seminiferous cycle-dependent expression of Gdnf mRNA in hamster seminiferous tubules (purple staining). In A and B, arrowheads indicate the border between high- and low Gdnf-positive areas. In C, SBA lectin staining (red fluorescence for acrosome staining; DAPI, blue in lower plate) using transverse sections of whole-mount stained seminiferous tubules (Gdnf signal, purple; upper plate) reveals the reduction in Gdnf expression between stages VII and VIII (inset indicates positive signals in Sertoli cells at stage VII). The changes are consistent with the immunohistochemical data (Fig. 2). (D–E) Section in situ hybridization analysis demonstrates high levels of Gdnf expression before spermiation in hamster testes (purple staining). Asterisks, non-specific signals in the acrosomes of round spermatids. Scale bars represent 100 µm in C and D, and 10 µm in E. (TIF) [file pone.0028367.s002.tif]

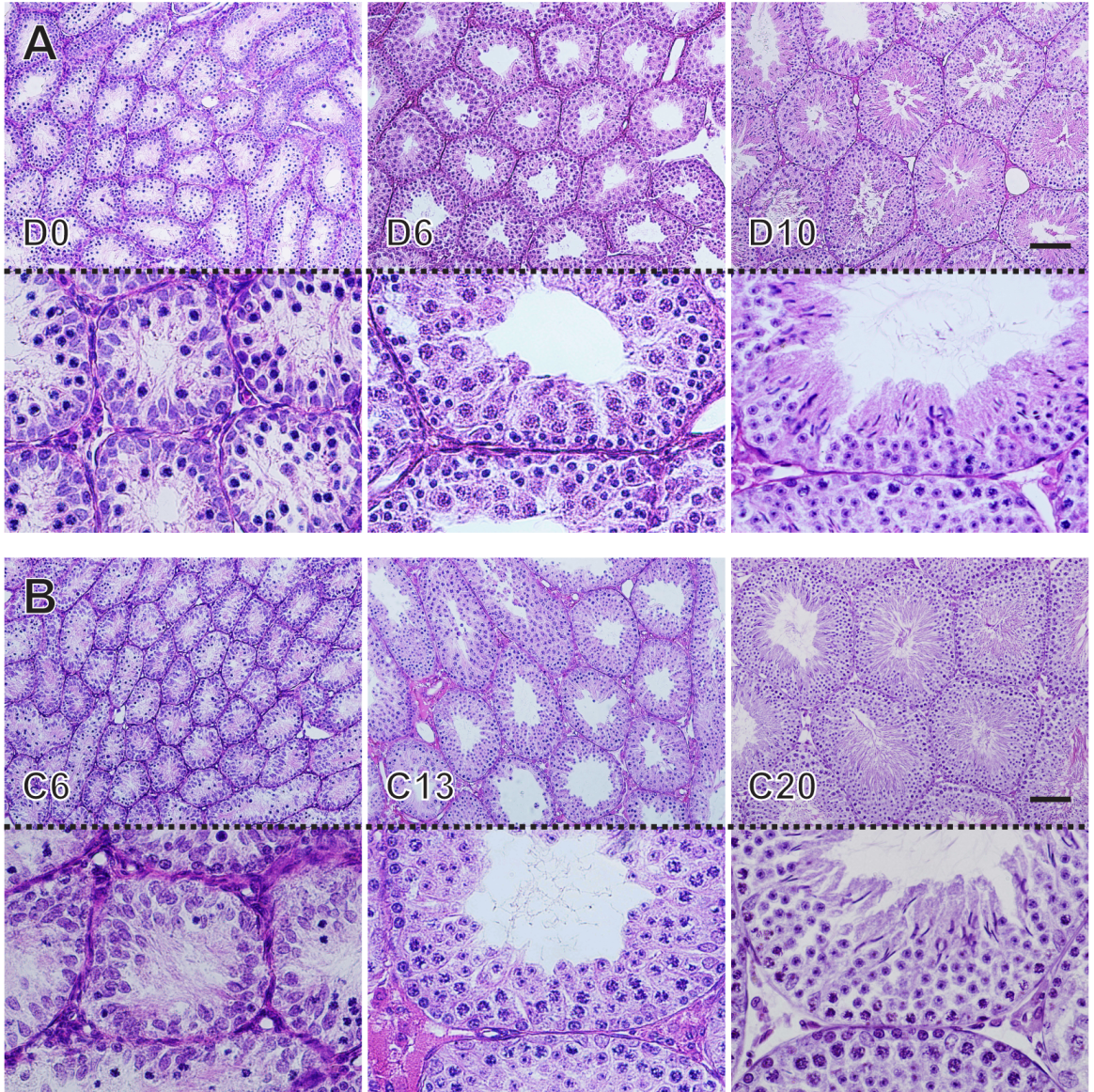

Supplement: Figure S3 — Histological analysis of seminiferous tubules in short photoperiod/low ambient temperature-induced “inactive” testes in adult hamsters. Adult hamsters (8-week-old) were exposed to a short photoperiod (6 h light, 18 h dark) and an ambient temperature of 23°C. After the testes reached the most “inactive” state in Week 13 of treatment (D0), half of the hamsters were maintained in an environment with an ambient temperature of 5°C (5°C group) for 6 (C6), 13 (C13), or 20 weeks (C20), respectively. The remaining hamsters were maintained in an environment with a stable ambient temperature of 23°C (23°C group) for 6 (D6) or 10 weeks (D10), respectively. After exposure to a short photoperiod for 13 weeks (D0), spermatogenic activity began to recover autonomously, with complete recovery observed within 10 to 20 weeks in both the 5°C (C20) and 23°C groups (D10). Scale bars represent 100 µm. (TIF) [file pone.0028367.s003.tif]

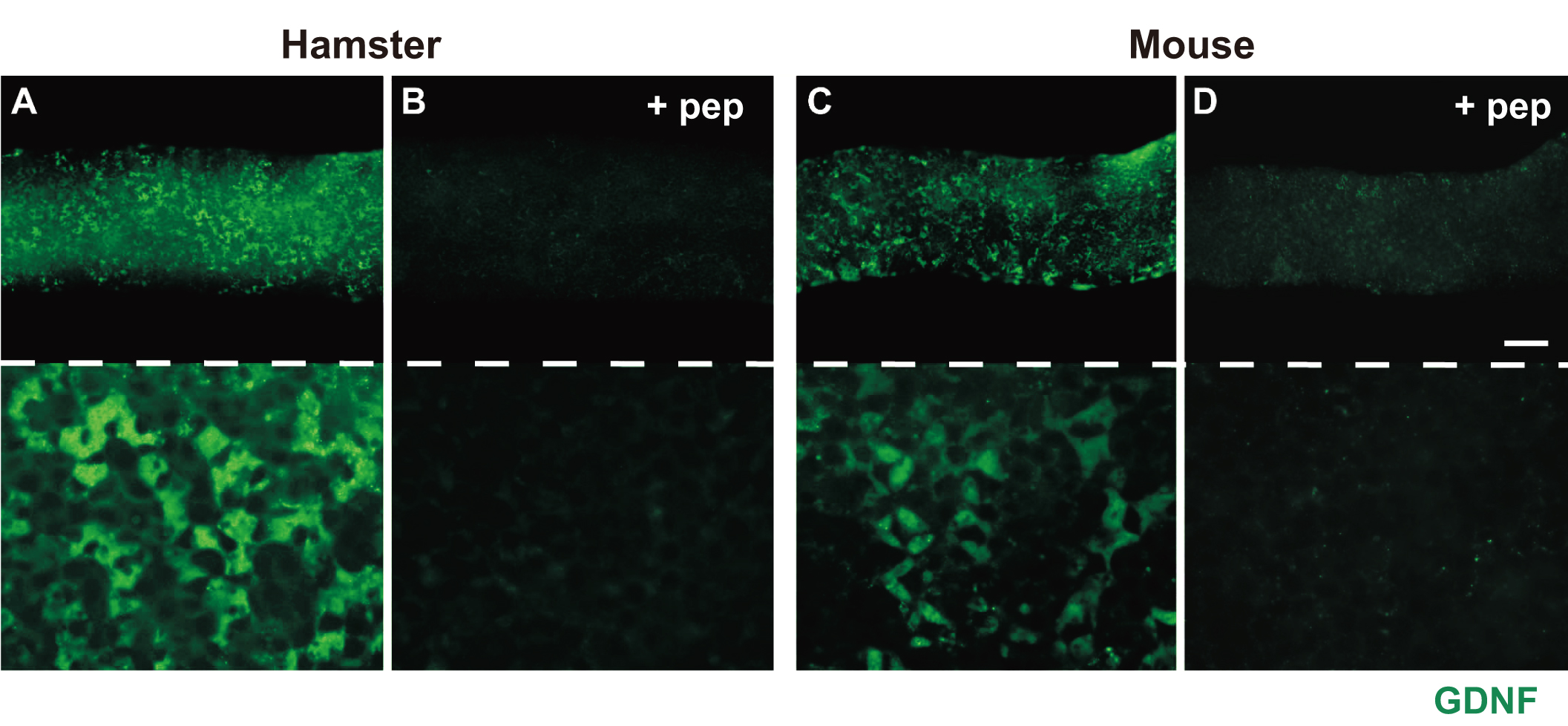

Supplement: Figure S4 — Negative controls for whole-mount anti-GDNF immunostaining (without permeabilization) of seminiferous tubules in hamsters and mice. Anti-GDNF antibody was pre-incubated with GDNF peptides (sc-328P; Santa Cruz Biotechnology) prior to use for whole mount immunostaining. The pre-treatment with GDNF peptides (+pep) greatly reduced GDNF-positive signals in both hamster (A, B) and mouse (C, D) samples. Each plate includes the inset panel showing a higher magnification image of upper panel. Scale bar represents 100 µm. (TIF) [file pone.0028367.s004.tif]

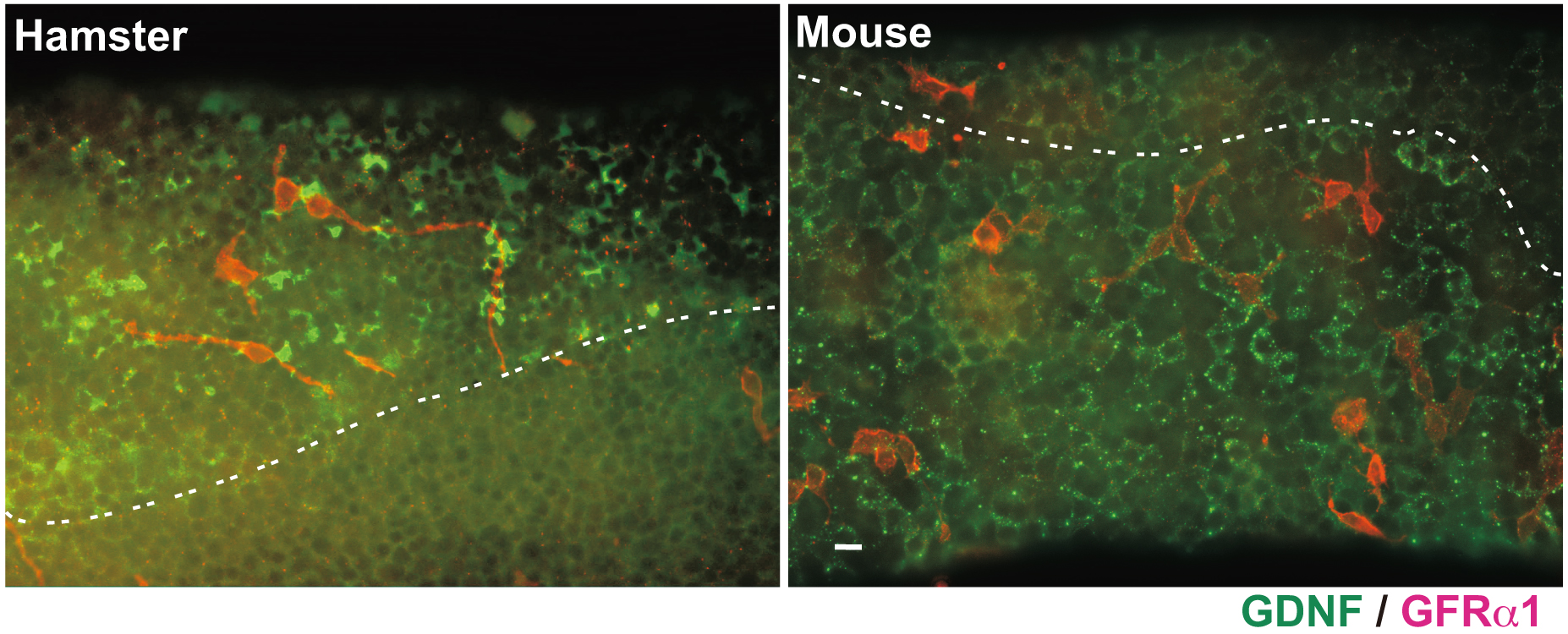

Supplement: Figure S5 — Comparative GFRα1/GDNF-double-staining images of the seminiferous tubules in hamsters and mice. Whole-mount immunostaining (without permeabilization) of seminiferous tubules showing GDNF-positive deposits (green) and GFRα1-positive spermatogonia (red) in the basal compartment of the seminiferous epithelia in hamsters (left) and mice (right). In each plate, the seminiferous tubule is shown at the same magnification. In the left plate, the lower edge of the tubule wall is missing due to the larger diameter of the seminiferous tubule in hamster than that of the mouse. Hamster GFRα1-positive cells are more slender in shape and lower in number than those in mouse GFRα1-positive cells. In both plates, dotted lines roughly indicate the border between GDNF-high and -low/negative areas of the seminiferous tubules. Scale bar represents 10 µm. (TIF) [file pone.0028367.s005.tif]
