# Supplementary material for: Cyclical and Patch-Like GDNF Distribution along the Basal Surface of Sertoli Cells in Mouse and Hamster Testes
Source: PLoS One. 2011 Dec 9;6(12):e28367. doi: 10.1371/journal.pone.0028367 (PMC3235125; doi:10.1371/journal.pone.0028367)
Supplement: Table S1 — Ratio of GDNF-positive seminiferous tubule in hamster testes at inactive and recovery period. (DOC) [file pone.0028367.s008.doc]

| Group **^b)^** | Ratio of GDNF-positive seminiferous tubules (%) ^c)^ | | | Total number of the seminiferous tubules |
| --- | --- | --- | --- | --- |
|  | **-** | **+** | **++** | counted |
| **Cont** | 50.0 ± 1.6 | 20.5 ± 2.1 | 29.5 ± 1.1 | 1076 |
| **D0** | 93.2 ± 1.1** | 6.8 ± 1.1** | 0.0 ± 0.0** | 222 |
| **D6** | 0.0 ± 0.0** | 58.4 ± 2.9** | 41.6 ± 2.9* | 561 |
| **D10** | 42.5 ± 3.8 | 19.5 ± 2.0 | 38.0 ± 3.8 | 460 |
| **C6** | 93.2 ± 1.5** | 6.8 ± 1.5** | 0.0 ± 0.0** | 236 |
| **C13** | 0.0 ± 0.0** | 56.6 ± 3.8** | 43.4 ± 3.8* | 470 |
| **C20** | 42.6 ± 5.0 | 22.0 ± 2.4 | 35.4 ± 4.1 | 582 |
| Mean ± S.E.M, *p<0.05, **p<0.01 (Dunnett Test, Two-tailed) **^d)^** | | | | |

**Table S1.** Ratio of GDNF-positive seminiferous tubule in adult hamster testes in photoregressed (D0) and hibernating (C6) states and during subsequent spontaneous recrudescence by prolonged exposure to inhibitory photoperiods (D6, D10, C13, C20) **^a)^**

1. The number of seminiferous tubules with or without GDNF-positive Sertoli cells were counted in the immunostained sections, and then the ratio of GDNF-positive seminiferous tubules were estimated in each group. The data in each group correspond to small circle graphs as shown in Fig. 3A.
2. Male adult (8wk old) hamsters (**Cont**) were exposed to a short photoperiod (6h light, 18h dark) at 23°C (normal room temperature). The testes photoregressed to the most “inactive” state at 13 wk of short photoperiod treatment (**D0**). A part of these “D0” hamsters were continuously maintained under a short period at 23°C for 6 wk (**D6**) and 10 wk (**D10**), while other groups were maintained under a short period at 5°C (low ambient temperature) for 6 wk (**C6**; hibernated state), 13 wk (**C13**) and 20 wk (**C20**).
3. All seminiferous tubules in the immunostained sections were assigned into three categories as follows: “-”, no GDNF-positive signals; “**+**”, some GDNF-positive Sertoli cells; “**++**”, almost all Sertoli cells are positive for GDNF in the tubule.
4. Dennett test was performed to determine statistically significant differences between control group and other groups.

(DOC)
